# Navigating through the Mundellian Trilemma: A dataset of four decades

**DOI:** 10.1016/j.dib.2019.104677

**Published:** 2019-10-17

**Authors:** Aleksandar Stojkov, Thierry Warin

**Affiliations:** a Ss. Cyril and Methodius University, Faculty of Law Skopje, Macedonia; b Skema Business School, Raleigh, NC, United States

**Keywords:** Trilemma, Financial openness, Monetary independence, Exchange rates

## Abstract

The Mundellian Trilemma has maintained its reputation of a sensible guiding framework regarding policy trade-offs among monetary independence, degree of exchange rate flexibility, and financial openness. This data article offers a unique access to explore the determinants and implications of different Trilemma configurations across 195 countries and over 58 years by providing macroeconomic, financial and policy choice data for 103 variables. The dataset has the potential to contribute to the investigation of the consequences of different Trilemma configurations by income group, size, and geographical position of the economies; to enrich international macroeconomics literature on the role of country idiosyncrasies in navigating through the Mundellian Trilemma, and to formulate policy-relevant conclusions.

**Table:** Specifications Table

Subject area	*Economics*
More specific subject area	*Monetary Policy, International Finance*
Type of data	*Excel-file*
How data was acquired	*Data extracted from five reputable sources.*
Data format	*Raw, filtered.*
Experimental factors	*Data allow empirical inquiries into the implications of a regime change in monetary policy, exchange rate arrangements or degree of financial openness.*
Experimental features	*This database could be used to understand the policy choices of national authorities in terms of exchange rate stability, monetary policy independence and financial openness.*
Data accessibility	*The Data is made available on Figshare at:*https://doi.org/10.6084/m9.figshare.8076509.v3

**Table:** 

**Value of the Data** • The dataset offers a unique access to more than four decades of relevant macroeconomic, financial and policy choice data for 103 variables. • Researchers and policymakers can evaluate and reassess policy choices made by the authorities of 195 countries and territories in terms of monetary policy independence, exchange rate regime, and degree of financial openness, the so-called Mundellian Trilemma. • The dataset enables researchers to follow and contribute to the most vibrant and passionate debates in international finance presently. They can investigate the validity of the celebrated Mundellian Trilemma in the context of highly volatile international capital flows and presumably very powerful Global Financial Cycles.

## 1 Data

The dataset with this article is a Stata file (dta).^1^ It provides macroeconomic data referring to 103 variables for 195 countries and territories. It combines data from five reliable sources: (1) three reputable sources of research-created data and (2) two official data sources (the World Economic Outlook database by the International Monetary Fund and the World Development Indicators by the World Bank). Considered separately, these databases are not entirely consistent in terms of country and time coverage. Our dataset homogenizes the different variables and allows for a further statistical treatment.

**Fig. 1:**
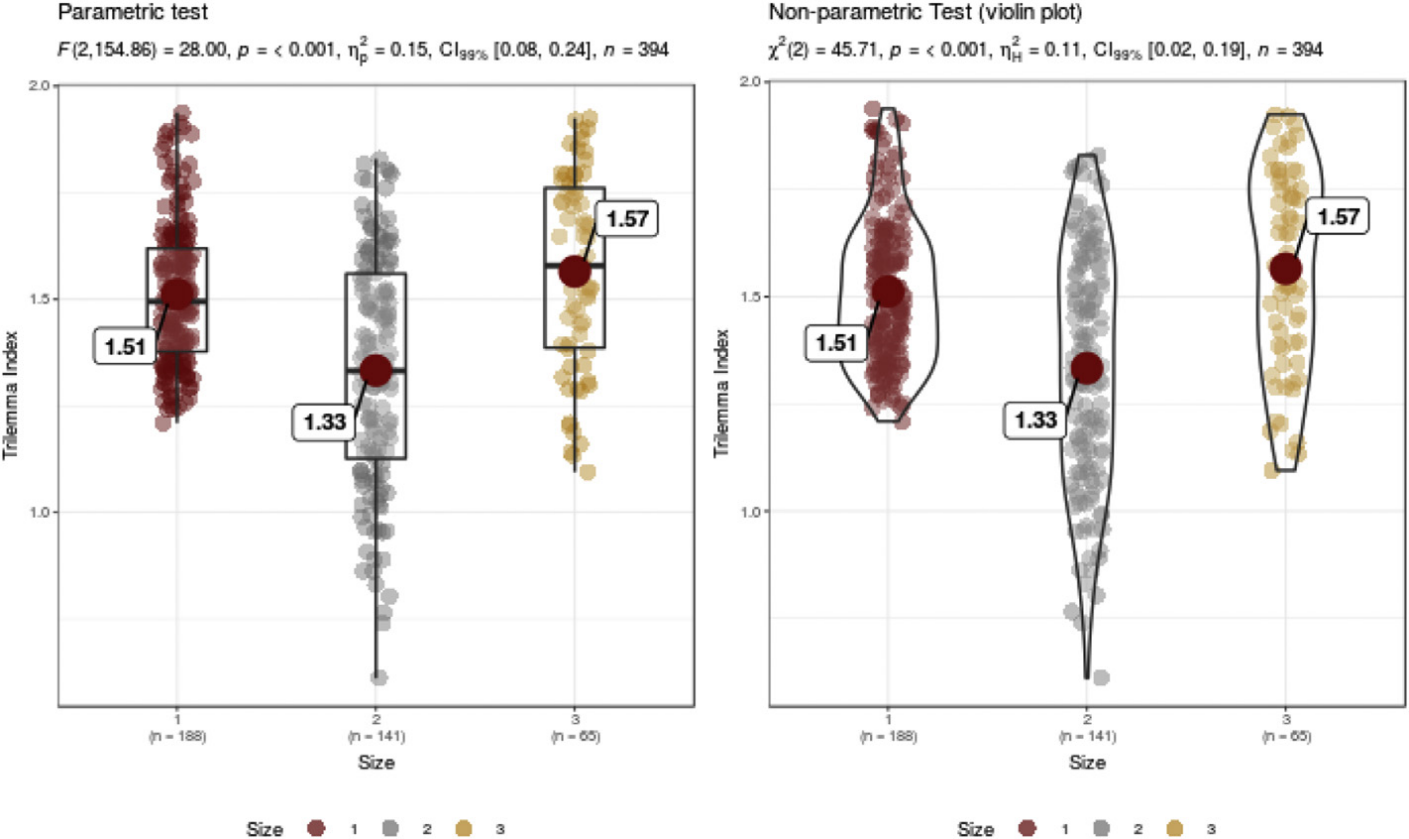
Differentiation of Trilemma indexes by the size of economies (small, medium-sized and large economies).

**Fig. 2:**
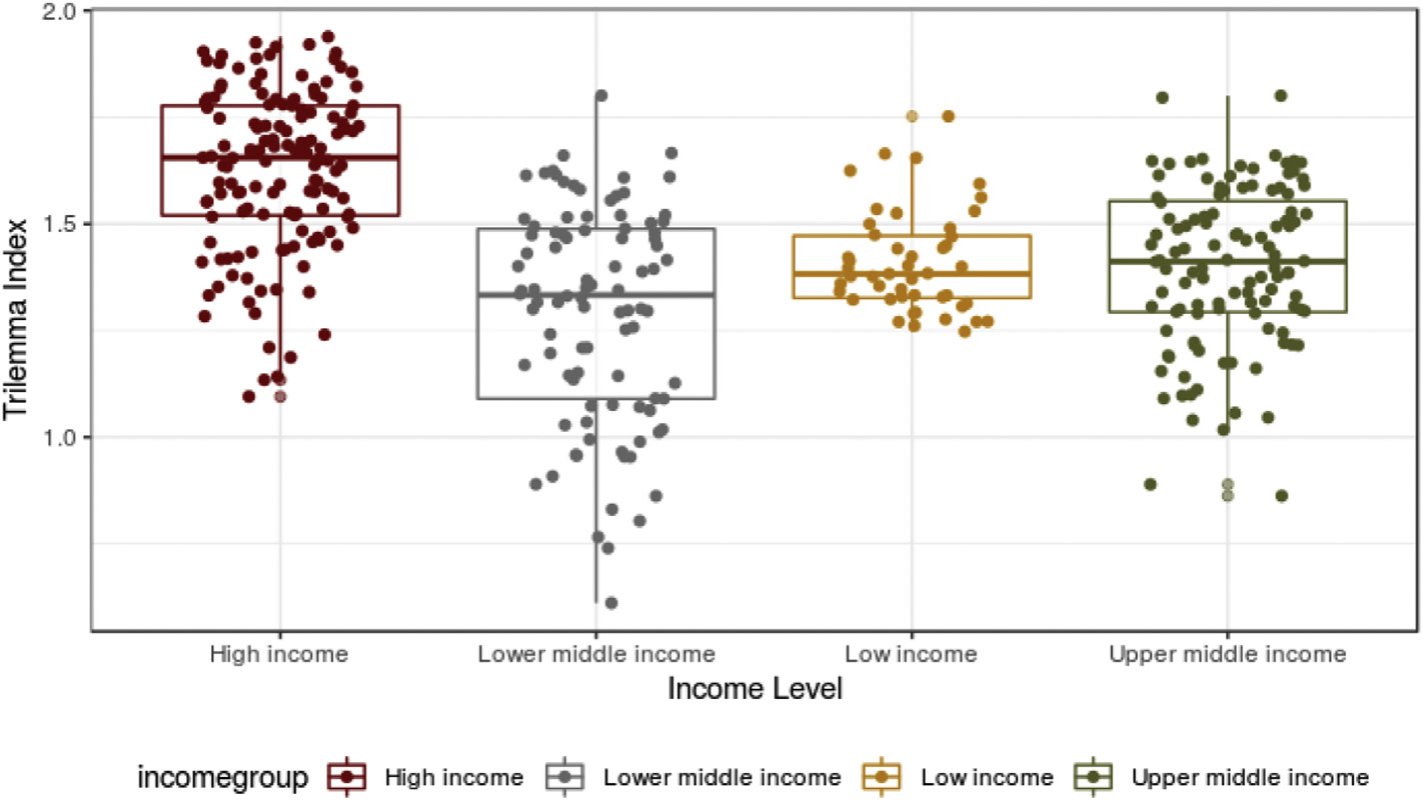
Differentiation of Trilemma indexes by income groups (high income, upper-middle income, lower-middle income and low income economies).

The first source of research-created data refers to the quantification of the degree of achievement of three important policy objectives by Ref. [1] for the period between 1960 and 2017. The second data source refers to (dis)aggregated data on inward and outward stocks of foreign capital, produced by Ref. [2] and referring to the 1970–2015 period. The third reputable source offers a dataset on financial development and financial structure of economies, publicly available by Refs. [[3], [4], [5]] referring to the 1960–2017 period. In order to encourage new research avenues, we differentiate the countries by the size of their economies (Fig. 1), by geographical regions, and by their level of economic development, proxied by the gross domestic product (GDP) per capita in purchasing power parity terms (Fig. 2).

The combined dataset refers to the period from 1960 to 2017 with missing data for 1960s and 1970s for several developing and transition countries. To the best of our knowledge, this is the first publicly available dataset that allows for a comprehensive analysis of policy choices and their implications regarding the monetary policy autonomy, exchange rate stability, and financial openness. Since not all policy options are simultaneously possible, this has also come to be known in international macroeconomics as the “monetary policy (or Mundellian) Trilemma.”

The description of the variables of the dataset is provided in Table 1. For a complete list of variables and the full dataset, please see the following link: www.mundelltrilemma.socialdata sciencelab.org.

**Table 1:** Structure of the dataset.

Type of variables	Number of variables	Examples of variables
A. Country Identification Variables	16	E.g., country code (i = 1, 2, …, 195), years spanning from 1960 to 2017 (t = 1, 2, .., 57), name of country or territory, dummy variables by income group (high-income, upper-middle income …), dummy variables by geographical regions (Europe, East Asia, Latin America …), and by size of economies (large, medium, and small).
B. Policy Variables	3	Exchange Rate Stability Index, Monetary Policy Independence Index, and Financial Openness Index, normalized in the range [0, 1] and updated until 2017.
C. Macroeconomic Variables	29	E.g., Gross domestic product (GDP), level, growth, per capita, current and constant prices, purchasing power parity terms; Gross domestic investment, Gross national saving, Inflation rate (end of year and annual average); Volume of exports and imports of goods and services, trade openness, general government budget data.
D. Variables on Inward and Outward Stocks of Foreign Capital	22	Inward and outward stocks of foreign direct investment (FDI), portfolio investment, and other investment; Net foreign asset position; International investment position; Exchange rates (end of year and annual averages).
E. Variables on Financial Development and Financial Structure	33	Domestic credit to the private sector to GDP; bank deposits to GDP; financial system deposits to GDP; bank concentration, profitability and liquidity ratios; stock market capitalization; public and private bond capitalization; number of listed companies; remittance inflows to GDP.
Total:	103	

The topic lays at a highly relevant meeting point of several strands of international macroeconomics literature, examining the international transmission of monetary policy shocks, the implications of different exchange rate arrangements, and the optimal degree of capital account liberalization. The dataset enables researchers to follow the most vibrant and passionate debates in international finance presently. They can contribute to the empirical literature on the validity of the Trilemma in the context of volatile net international capital flows and presumably very powerful Global Financial Cycle.

**Fig. 3:**
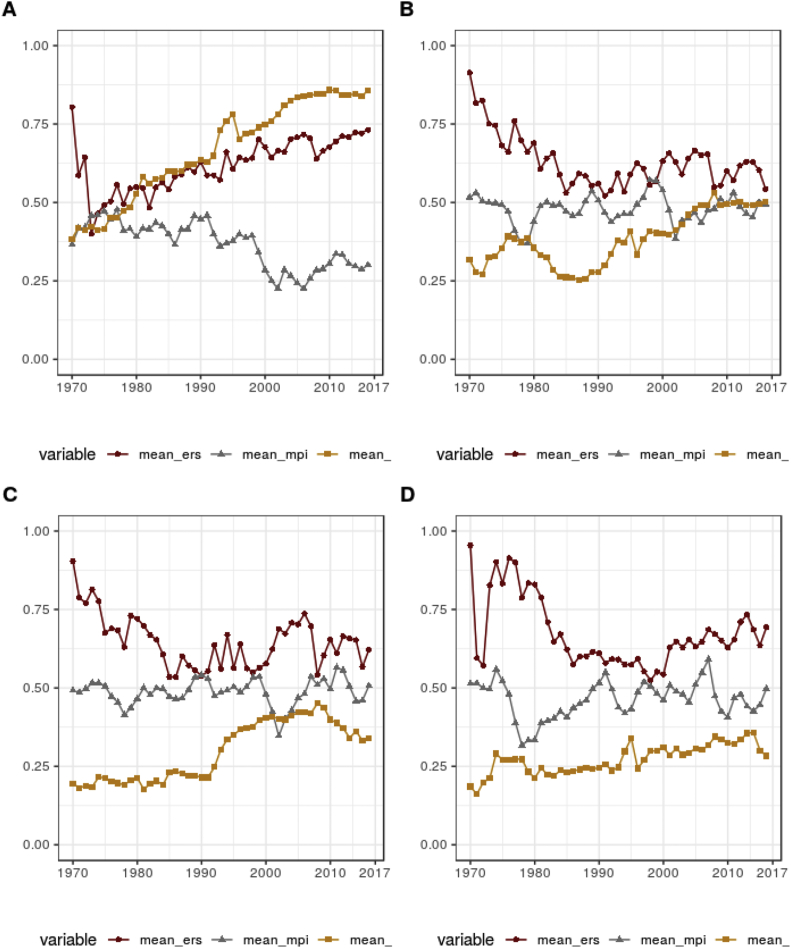
Summary statistics of the trilemma variables. *Note:* A, B, C and D correspond respectively to high-income, upper-middle income, lower-middle income and low-income countries. Data refer to the full sample of 5994 country-year observations, i.e. 195 countries over the period from 1960 to 2016 with a lot of missing observations for the developing and transition countries during the 1960s and 1970s. The calculations take into account only the trilemma configurations when data for all three variables for a particular country-year observation is available. *Source:* Constructed by using data by Ref. [1], and updated until 2017.

Finally, the evolution of the Trilemma variables (monetary policy independence, exchange rate stability, and degree of financial openness), which are key to the very vibrant debate, is presented in Fig. 3.

## 2 Experimental design, materials and methods

Data from various official sources were manually acquired [[1], [2], [3], [3], [4], [5], [6], [7]]. Official country names were initially harmonized, ordered by income groups and by year, and given codes in order to ensure consistency. Data from other sources was allocated sequentially to each of the 11,310 country-year observations (*N* = 195 countries; *t* = 58 years) in the most comprehensive database compiled by Ref. [1] that spans from 1960 to 2017. The Microsoft Excel functions of sorting, filtering and true/false logical statements have been extensively used to warrant compatibility of other data with the most comprehensive database. To the best of our knowledge, this provides a unique dataset for examining the determinants of different policy choices and their macroeconomic implications.

## Footnotes

1 The dataset is available on Figshare at: https://doi.org/10.6084/m9.figshare.8076509.v3.
